# Assessment of Antioxidant Activity and Neuroprotective Capacity on PC12 Cell Line of *Frankenia thymifolia* and Related Phenolic LC-MS/MS Identification

**DOI:** 10.1155/2016/2843463

**Published:** 2016-10-18

**Authors:** Rim Ben Mansour, Wided Megdiche Ksouri, Stéphanie Cluzet, Stéphanie Krisa, Tristan Richard, Riadh Ksouri

**Affiliations:** ^1^Laboratory of Aromatic and Medicinal Plants, Center of Biotechnology, Technopark of Borj-Cedria (CBBC), BP 901, 2050 Hammam-Lif, Tunisia; ^2^University of Bordeaux, ISVV, GESVAB EA3675, 33882 Villenave d'Ornon, France

## Abstract

This work aimed to investigate the richness of a Tunisian xerohalophyte* Frankenia thymifolia* aerial and root parts on phenolics and to evaluate the antioxidant and neuroprotective properties of this medicinal species. After fractionation using increasing and different solvent polarities, results displayed five fractions, where ethyl acetate (EtOAc) shoot and root fractions possess considerable total phenolic contents (221 and 308 mg of GAE/g of E, resp.) related to their important antioxidant activities such as ORAC (918 and 713 mg of TE/g of E), DPPH (282 and 821 mg of TE/g), and ABTS (778 and 1320 mg of TE/g) tests. Then, the identification of the main compounds by HPLC-DAD-ESI-MS and neuroprotective property of the most active fraction EtOAc were assessed. A total of 14 molecules were identified, which have been described for the first time in* F. thymifolia*. The major compounds identified were pinoresinol and kaempferol glycoside in aerial parts and gallic acid and ellagitannin in roots. Neuroprotective capacity against *β*-amyloid (A*β*) peptide induced toxicity in PC12 cells of EtOAc fraction showed a significant protective activity at lower concentration (25 and 50 *µ*M). The relevant antioxidant and neuroprotective activities of* F. thymifolia* EtOAc fraction corroborated their chemical compositions.

## 1. Introduction

Many lifestyle factors endorse health of the nervous system in trouble by imposing a mild stress on neural cells and demand for phytotherapeutic agents is growing, in view of synthetic drugs that are believed to have certain side effects such as dry mouth, tiredness, anxiety or nervousness, dementia, and pseudodementia [1]. Effects of these synthetic drugs have caught attention from research bases and industries towards natural herbal resources [2]. In addition to their antioxidant and several health promoting activities, natural bioactive compounds including phenolics, flavonoids, alkaloids, terpenoids, lignans, and saponins have potential properties to modulate neuronal function, protective mechanism against neurodegeneration, and memory enhancing properties and attenuate the damaging effects of reactive oxygen species (ROS). Many factors are known to play a direct role in the initiation of neurodegeneration; free radical formation by ROS is the main causative factor [3]. Excess of ROS in the body can lead to cumulative damage in cellular structures, resulting in so-called oxidative stress [4]. Neurons and brain cells are particularly vulnerable to free radicals, and oxidative stress is one of the main causative factors in the etiology of a number of late onset disorders [1, 5]. In addition, oxidative stress seems to mediate *β*-amyloid peptide toxicity by free radical production, suggesting a pathophysiological relation between A*β* and imbalance between reactive oxygen production and protective system [6]. Neuronal cells oxidative damage may be a source of endogenous production of ROS, and amyloid beta (A*β*) peptide may increase ROS production causing further impairment of cellular structure function in brain [7, 8]. A*β* is the main component of senile plaques and is highly involved in the progression of neurodegenerative diseases and impairment activity of several complexes of mitochondrial respiratory chain in neurons and astrocytes [8]. Finding molecules such as phenolics play a major part in inhibition of propagation of oxidative chain reaction and in maintaining the brain's chemical balance by acting upon the function of receptors for the major inhibitory neurotransmitters [1]. These compounds prevent aggregation and attenuate A*β*-induced toxicity, protein oxidation, and apoptosis in primary hippocampal cultures [9, 10]. In Tunisia, considerable diversity of folkloric medicinal halophytes (spontanoeus plants) is known for their ability to withstand and quench these toxic ROS and for their strong biological properties, sometimes exceeding many natural antioxidants from medicinal cultivated species (glycophytes). Among them,* Frankenia thymifolia* belonging to Frankeniaceae family is an endemic species from North Africa. Wided et al. [11] reported that* F. thymifolia* exhibit high polyphenol, flavonoid and tannin contents, and antioxidant and antibacterial activities. Phytochemical studies on the genus* Frankenia* and the information on the chemical composition of* F. thymifolia* are still scarce. There are only studies that address the identification of pinoresinol 4-sulfate, lignan sulfate, and two aromatic compounds (1,2,3,4,5,7-hexamethoxynaphthalene and 4,5-dimethoxy-3-hydroxybenzoate methyl) in* F. thymifolia* Desf. [12].

Due to the importance of identifying new compounds with interesting antioxidant and biological activities, we describe the optimization of aerial parts and roots fractionation of* F. thymifolia* using solvents with increasing polarity. The crude extracts and obtained fractions were evaluated in terms of their antioxidant properties through different antioxidant tests such as DPPH, ABTS, metal chelating activity (MCA), and ORAC. In addition, the neuroprotective activity against *β*-amyloid peptide on PC12 cell line and the investigation of phytochemical composition of EtOAc fractions by using HPLC-DAD-MS/MS were assessed.

## 2. Materials and Methods

### 2.1. Plant Material and Extraction

*F. thymifolia* was collected during the vegetative stage in March 2014 from Borj-Cedria (latitude: 36°46′N and longitude: 10°39′E) at 30 Km to Tunis. This halophyte was identified at the Biotechnology Centre (CBBC, Technopark of Borj-Cedria), and a voucher specimen [PLM52] was deposited at the Herbarium of the Laboratory of Medicinal and Aromatic Plants at the CBBC. After air drying, aerial parts and root extracts were obtained by magnetic stirring of 150 g of matter powder with 1500 mL methanol 80% for 2 h; then the filtrate is evaporated using a rotary evaporator. The obtained filtrate is first extracted with hexane followed by dichloromethane, ethyl acetate, and finally butanol. The different phases are separated by a separatory funnel.

### 2.2. Total Phenolic Contents

Total phenolic contents (TPC) of aerial part and root extracts were determined by the Folin-Ciocalteu colorimetric method [13] adapted to 96-well plate. To 20 *µ*L of extract (1 mg/mL), 100 *µ*L of Folin-Ciocalteu's reagent was added. After 2-3 min, 80 *µ*L of sodium carbonate (75 g/L) solution was added. After 1 h, the absorbance was measured at 765 nm. The TPC was expressed as mg of gallic acid equivalent per g of extract (mg of GAE/g of E). Experiments were analyzed at least three times and with triplicate samples.

### 2.3. Radical Scavenging Assay

Radical scavenging ability against DPPH radical was measured as described by Blois [14]. A volume of 50 *µ*L of each sample (1 mg/mL) was mixed with 150 *µ*L of 200 *µ*M methanolic solution of DPPH in a 96-well plate. The plate was allowed to stand in dark for 20 min. The absorbance was measured at 520 nm.

The scavenging activity of the extracts on ABTS radical cation was estimated according to the method of Re et al. [15]. Briefly, 250 *μ*L of the diluted ABTS^+^ solution was added to 10 *μ*L of extracts at the concentration of 1 mg/mL (or Trolox). Six minutes after initial mixing, the absorbance was measured at 734 nm at 30°C. Results were expressed as mg of TE/g of E. All samples were analyzed in triplicate in at least three different experiments.

### 2.4. Metal Chelating Activity (MCA)

The chelating activity of the extracts for ferrous ions Fe^+2^ was measured according to the method of Dinis et al. [16]. A volume of 80 *μ*L of deionized water and 40 *μ*L of FeSO_4_ (0.2 mM) were added to extract (40 *µ*L, 1 mg/mL) and mixed in 96-well microplate. The reaction was initiated by addition of 40 *μ*L of ferrozine (2 mM). The absorbance of Fe^2+^-ferrozine complex was measured at 562 nm after 10 min. EDTA was used as standard and results were expressed as mg of EDTA per gram of extract (mg of EDTA/g of E). All samples were analyzed in triplicate in at least three different experiments.

### 2.5. ORAC Assay

The procedure was modified from the method described by Ou et al. [17], using Trolox as a control standard. The ORAC assay was carried out in black round bottom 96-well microplates (Costar) and absorbance was measured with an automated plate reader (Fluostar Optima, BMG Labtech). All the samples (extracts, fluorescein, and AAPH) were diluted in 75 mM phosphate buffer (pH 7.4). Thirty microliters of each extract (1 mg/mL) or phosphate buffer (blank) was mixed with 180 *μ*L of fluorescein solution (117 nM final concentrations) and incubated for 5 min at 37°C. A volume of 90 *µ*L of AAPH solution (40 mM final concentration) was added and fluorescence was immediately monitored using 485 nm excitation and 520 nm emission wavelengths at 1 min intervals for 70 min. The antioxidant capacities of the extracts were expressed as mg of Trolox equivalent per g of extract (mg of TE/g of E). All samples were analyzed in quadruplicate and at least in three different experiments.

### 2.6. Cell Culture and MTT Assay of Shoot and Root EtOAc Fraction

Pheochromocytoma-derived PC12 cells (ATCC, Manassas, VA, USA) were maintained routinely in DMEM-Glutamax supplemented with 15% horse serum, 2.5% fetal bovine serum, and 1% penicillin/streptomycin antibiotics at 37°C in humidified atmosphere of 5% CO_2_/50% air. Cells were plated at a density of 30.000 cells per well in 96-well plates and incubated at 37°C for 24 h. Then, the cells were treated with 5 *µ*M of A*β*_(25–35)_, with or without extracts at 25, 50, 100, 200, and 300 *µ*M in serum-free culture medium. After 24 h of incubation, cell viability was determined by the conventional MTT reduction assay. Cells were treated with MTT solution (0.5 mg/mL) for 3 h at 37°C. The dark blue formazan crystals formed in viable cells were solubilized with DMSO for 0.5 h. The absorbance was measured at 595 nm with a microplate reader (Dynex, USA). Results were expressed as the percentage of MTT reduction in relation to the absorbance of control cells at 100%. All data represent the average of four tests.

### 2.7. HPLC-DAD-ESI-MS

Lyophilised EtOAc fractions were dissolved in 50% methanol and chromatographed using HPLC-DAD-ESI-MS system. The chromatography apparatus was Agilent 1200 from Agilent Technologies (Santa Clara, CA, USA). The EtOAc fractions were analyzed at 25°C with a 250 × 4 mm i.d. 5 *μ*m, Prontosil 120-5-C18-AQ reverse phase column (Bischoff, Leonberg, Germany). Water, 0.1% HCOOH (solvent A), and acetonitrile 0.1% HCOOH (solvent B) were used as mobile phases. The gradient elution program was as follows (v/v): 0 min 1% B, 0.4 min 1% B, 2 min 10% B, 6 min 35% B, 7 min 50% B, 8.8 min 70% B, 10.8 min 92% B, 11 min 100% B, and 12 min 100% B, followed by 10 min for reequilibration. The optimum values of the ESI-MS parameters were as follows: capillary voltage, −4.7 kv; drying gas temperature, 350°C; drying gas flow, 10 L/min; nebulising gas pressure, 35 psi. LC/MS accurate mass spectra were recorded across the range 150–2000* m/z*. The detection wavelengths were set at 280 and 360 nm. LC-ESI-MS analyses were carried out in the negative ion mode. This HPLC was coupled to Esquire 3000+ ion trap mass spectrometer using ESI source from Bruker Daltonics (Billerica, MA, USA).

### 2.8. Statistical Analysis

Results are expressed as means ± standard deviation of three replicates. Multiple sample comparison was performed using the Statgraphics Plus program version 5.1 for windows. Analysis of variance (ANOVA) followed by Duncan's multiple comparison test was used. Whenever ANOVA could not be used, Kruskal-Wallis test was applied after checking for normal distribution of the groups and homogeneity of variances. The level of significance was *P* < 0.05. In order to compare the different values of antioxidant activities obtained in our extracts after all types of antioxidant measurement, the Pearson correlation test was used. Alternatively, the results were analyzed by GraphPad Prism 5.03 for Windows (GraphPad Software, San Diego, CA, USA).

## 3. Results and Discussion

### 3.1. Total Phenolic Content

Methanolic crude extract and five fractions (hexane, dichloromethane, ethyl acetate, butanol, and water) of* F. thymifolia* aerial parts and roots were analyzed for total phenolic contents (TPC) and reported in Table 1. TPC were higher in root than in aerial parts and varied significantly as function of solvent. The highest recovery of TPC was observed in ethyl acetate fraction reaching up to 221 and 308 mg GAE/g in aerial part and root, respectively, while the lower recovery was observed in hexane fraction (13 mg of GAE/g for aerial parts and 55 mg of GAE/g for roots). However, given the low polarity of EtOAc (polarity index 4.4) when compared with butanol, methanol (5.1), or water (polarity index 9), it seems logical to suppose that the highest recovery of TPC by using EtOAc was presumably due to its high molecular weight (88 g/moL) which enable it to easily extract about the same molecular weight following the concept “like dissolves like.” Fernandes de Oliveira et al. [18] reported that ethyl acetate phase of* Sidastrum micranthum* (Malvaceae) had the highest content of TPC (177.44 mg of GAE/g) compared to other phases such as butanol, water, dichloromethane, and hexane. These data highlight that the solubility of the phenolics is governed by solvent polarity, degree of polymerization of phenolics, and the part of the plant used [19].

### 3.2. Antioxidant Activities

In the objective of choosing the adequate solvent for antioxidant capacity, DPPH, ABTS, ORAC, and MCA tests were used to evaluate the electron transfer and the hydrogen atom transfer capacity of each* F. thymifolia *fraction. Results depicted that root fractions exhibit better performances than aerial part against DPPH radical. Moreover,* F. thymifolia *was found to possess a significant variability in its inhibitory activity against this radical as a solvent function (Table 1). The EtOAc fraction is still the most active with 282 and 821 mg  of TE/g of E for aerial parts and roots, respectively. Fernandes de Oliveira et al. [18] also found that the EtOAc fraction of* Sida rhombifolia* (Malvaceae family) exhibits the highest antiradical activity as compared to hexane, dichloromethane, butanol, and water fractions. Anagnostopoulou et al. [20] showed also that the higher radical scavenging activities were found for the ethyl acetate fraction of sweet orange peel (*Citrus sinensis*). In the same context, Cakir et al. [21] showed that ethyl acetate fraction exhibited the highest DPPH radical scavenging activity from the aerial parts of* Hypericum hyssopifolium* L. These data are in agreement with previous study of Saada et al. [22], which showed that EtOAc fraction of the halophyte* Retama raetam* (Fabaceae family) exhibits the highest total phenolic compounds and antioxidant activity.

Results consigned in Table 1 revealed also that scavenging ability of ABTS radical of the aerial part fractions is less active than root fractions. Independent of the organ, EtOAc fraction displayed the higher ability to quench ABTS^+*∙*^ free radical in aerial part and root (778 mg of TE/g of E; 1320 mg TE/g of E, resp.). Total phenolics generally correlate with antioxidant capacities as measured by the ABTS or DPPH methods [23].

Measuring the ability of oxygen radical absorbance capacity (ORAC) was widely used in the field of antioxidants and oxidative stress. The antioxidative potential of different fractions evaluated by ORAC indicated that EtOAc fraction is the most active (918 and 713 mg TE/g in aerial parts and roots, resp.). Though, hexane fraction of aerial parts showed very low peroxyl radical scavenging activity, which was around 15 times lesser than EtOAc fraction (Table 1). In this context, Surget et al. [24] observed that EtOAc fraction of the halophyte* Salicornia ramosissima* showed high ORAC activity. Lizcano et al. [25] showed that ABTS and ORAC antioxidant activity values increased with increasing polyphenol content of the plant extracts.

The metal chelating activities (MCA) of the* Frankenia *fractions were monitored in order to evaluate the ability to inhibit interactions between metals and lipids. Overall, iron chelating activities were higher in aerial part fractions than in root. EtOAc fractions showed the higher activity (39 and 22 mg EDTA/g for aerial parts and roots, resp.). High iron levels may act catalytically to produce reactive oxygen species (Fenton reaction), with a negative impact on the structure and function of cells [26]. Iron can promote the formation of hydroxyl radicals and decompose lipid hydroperoxides into highly reactive lipid alkoxyl and peroxyl radicals, which perpetuate the chain reaction of lipid peroxidation [26, 27]. Many reports showed in this context the influence of the solvent on the capacity of extracts to chelate Fe^2+^ [28].

On the other hand, positive and strong correlations (*r* > 0.9) were found between TPC and each of the antioxidant assays (DPPH, ABTS, ORAC, and MCA) used in both aerial part and root of* F. thymifolia* (Table 3), which indicated that polyphenols in* F. thymifolia* extracts are largely responsible for the antioxidant activities. However, no correlation was found for ORAC and MCA tests in root and aerial part, respectively. A study performed by Babbar et al. [29] showed that phenolic contents alone are not fully responsible for the antioxidant activity of plants. Other constituents such as ascorbate, reducing carbohydrates, tocopherols, carotenoids, terpenes, and pigments as well as the synergistic effect among them could possibly contribute to the metal chelating activity.

### 3.3. HPLC-DAD-ESI-MS

**Figure 1:**
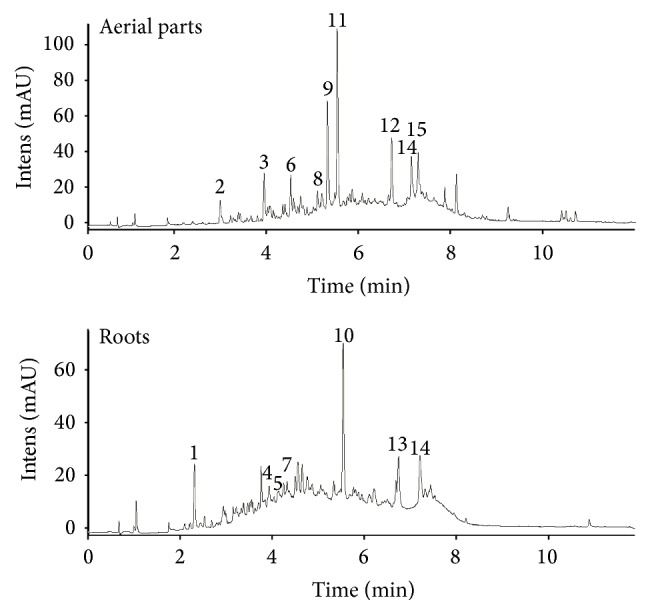
HPLC-DAD-ESI-MS base peak chromatograms in negative ion mode and UV at 280 nm for the ethyl acetate fraction of aerial parts and roots of* F. thymifolia*.

In next study, identification of the predominant compounds of EtOAc fractions from aerial and root parts was assessed by HPLC-DAD-ESI-MS in negative mode (Figure 1). For the first time, the methodology used in this work allowed us to identify 14 new phenolic compounds. All of them have not been described so far in* F. thymifolia*. The compounds detected in this work were tentatively characterized by means of MS data, together with the interpretation of the observed MS-MS spectra in comparison with those found in the literature and public databases (Table 2).

Compound** 1** at* m/z* 169 corresponds to gallic acid. It was identified with fragmentation ion at* m/z* 125 ([M − H − 44]^−^) corresponding to the loss of carboxylate.

Analysis revealed the presence of another phytochemical with antioxidant properties, hydroxytyrosol (compound** 2**) in the negative ESI-mode with ions at* m/z* 153, and a single diagnostic fragment at* m/z* 123 corresponding to the formal loss of a formaldehyde unit [30]. Compound** 3** (RT 3.9 min) shows a profile with [M − H]^−^ at* m/z* 137, which, under MS/MS conditions, yields fragments at* m/z* 93 characteristic of this compound that is assigned as* p*-hydroxybenzoic acid [31]. In the negative mode, hydroxybenzoic acids produced deprotonated [M − H − 44]^−^ fragment ion via loss of CO_2_ group from the carboxylic acid moiety [32]. The UV spectra of the hydroxybenzoic acids were quite relevant to their chemical structure.

The heterocyclic ring fragment pathways of flavan-3-ols are through quinone-methide, retro-Diels-Alder, and heterocyclic ring fission [33]. The protonated ion at* m/z* 609 (peak** 4**) produced MS fragment ions at* m/z* 483 through heterocyclic ring fission (loss of C_6_H_6_O_3_),* m/z* 441 through the retro-Diels-Alder fission (loss of C_7_H_6_O_3_),* m/z* 423 through the same fission following the loss of one H_2_O, and* m/z* 305 through quinone-methide [33, 34]. Hence, compound** 4** was assigned as prodelphinidin B-4. This compound was previously isolated and identified as the main flavonols in tea [35]. However, it was reported here for* F. thymifolia* for the first time.

Compound** 5** (Rt 4.2 min) exhibited [M − H]^−^ ion at* m/z* 635 with *λ*_max_ at 280 nm and fragment at* m/z* 465 corresponding to loss of gallic acid* m/z* 170 [36]. Therefore, this compound was identified as trigalloyl hexoside.

Peak** 6** that gave [M − H]^−^ ions at* m/z* 333 corresponds to posthumulone which yielded the two fragment ions at* m/z* 289 and* m/z* 265 corresponding, respectively, to the characteristic losses of propyl group [M-H-C_3_H_7_] and prenyl group [M − 68] [37].

Peak** 7** (Rt 4.5 min) at* m/z* 449 is assigned as luteolin 7-*O*-glucoside; the* m/z* 449 ion generated fragment ions at* m/z* 287, corresponding to luteolin aglycone in structure; and* m/z* 431 corresponds to loss of H_2_O ([M − H − 18]).

Quercetin-3-*O*-galactoside (hyperoside, compound** 8**) was identified by MS/MS analysis in neutral loss of 162 amu (loss of tetrahydroxylated hexose) [38]. Its ion fragments were observed at* m/z* 301 in full scan mode. The observed fragment ions at* m/z* 178 and 151 correspond to quercetin fragmentation pattern [39].

Compound** 9** found in aerial parts is speculated as pinoresinol (lignan) with* m/z* 357 and it produced the ion at* m/z* 329 corresponding to loss of CO [40]. In this context, Harkat et al. [12] previously identified pinoresinol 4-sulfate (sulfated lignan) in* F. thymifolia* roots.

Compound** 10** corresponding to unknown ellagitannin is a phenolic compound with sugar core exhibiting typical features in LC-MS analysis: loss of gallic acid moieties (152 amu), hexahydroxydiphenyl glucose unit (HHDP-glucose, 482 amu), and repetitive loss of water molecules and tendency to form double-charged ions [41]. UV/visible spectrum shows *λ*_max_ at the range of 290–320 nm. Several ellagitannins are typically detected in this MS spectrum [42].

Furthermore, compound** 11** had [M − H]^−^ at* m/z* 447 with fragment at* m/z* 285 (loss of 162 amu: hexose moiety) and was identified as kaempferol 3-*O*-glucoside [43].

Compound** 12** ([M − H]^−^ at* m/z* 312) has been assigned as feruloyl glycoside. MS/MS spectrum of this compound has shown the characteristic product ion at* m/z* 135 corresponding to the feruloyl moiety and a fragment at* m/z* 178. Identity of the compounds was verified by characteristic UV spectra showing *λ*_max_ at 290–320 nm.

Compound** 13** remains unidentified. No data corresponding to its mass spectra in the literature and databases was reported. However, peak** 14** has fragment ion* m/z* 80 corresponding to sulfate and UV-vis spectrum shape with *λ*_max_ at 290 and 360 nm proposed as sulfated flavonoid.

Compound** 15** is identified as eriodictyol hexoside; according to the characterization of [M − H]^−^ ion at* m/z* 449, it is a flavonoid derivate. All these flavonoids cited were not observed previously in* F. thymifolia*.

### 3.4. Neuroprotective Activity of EtOAc Fraction

**Figure 2:**
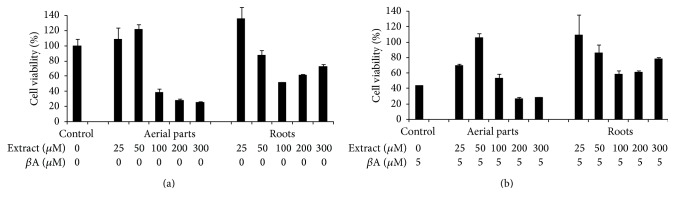
Cytoprotective effects of EtOAc extracts (a) and neuroprotective activity (b) on A*β*-induced toxicity in PC12 cell line. The experiment was repeated three times.

Due to the strong TPC and the antioxidant capacities of EtOAc fractions, the protective effect of these fractions against A*β*-induced toxicity in PC12 cell line was further investigated. First, the cytotoxic potential of each EtOAc fraction on PC12 cells was measured with the MTT assay. EtOAc fractions of both organs were not cytotoxic at the concentrations of 25 and 50 *µ*g/mL (Figure 2(a)). Treatment of PC12 cells with 5 *µ*M A*β*_25–35_ reduced cell viability about 40% of control (Figure 2(b)). Induction of cytotoxicity by A*β*_25–35_ at 5 *μ*M was then used for all subsequent experiments to evaluate the protective effect of the species.* F. thymifolia *fractions completely reversed the toxic effect of A*β*_25–35_ (at 25 and 50 *µ*g/mL for aerial parts and roots, resp.), indicating a significant neuroprotective effect of* F. thymifolia* EtOAc fractions. Nevertheless, results displayed a competitive effect at higher concentrations (>100 *µ*g/mL) between the toxicity of* F. thymifolia* on PC12 cells and the protective effect against A*β*-induced toxicity. In fact, this neuroprotective activity is mainly correlated to the nature of phenolic compounds in EtOAc fractions. In this context, several reports have demonstrated that gallic acid and ellagitannins, two major compounds identified in roots, inhibit efficiency *β*-amyloid (A*β*) peptide aggregation* in vitro* [44], while kaempferol 3-*O*-glucoside [45] and* p*-hydroxybenzoic acid [10] were found to possess moderate inhibitory effect on A*β* aggregation. Zeng et al. [46] showed that hyperoside significantly inhibited A*β*_25–35_-induced cytotoxicity and apoptosis by reversing A*β*-induced mitochondrial dysfunction, including mitochondrial membrane potential decrease, reactive oxygen species production, and mitochondrial release of cytochrome c.

## 4. Conclusion

*F. thymifolia* species have a high content of phenolic compounds and a good antioxidant and neuroprotective activities; therefore they can be used to treat several diseases in which there is an increase in free radical production. Moreover, the nature and polarity of solvent had a significant impact on the phenolic content and antioxidant activity. Overall, the EtOAc fraction of the halophyte* F. thymifolia *contained the highest levels of phenolic content and antioxidant activities. It has been established also that HPLC-DAD-ESI-MS is a powerful analytical technique for the separation and detection of phenolics in* F. thymifolia*. The obtained data indicate qualitatively that EtOAc fraction of* F. thymifolia* is an abundant source of bioactive phytochemicals and it could explain the antioxidant and neuroprotective capacities of this species.

## Figures and Tables

**Table 1:** Total phenolic content (TPC) and antioxidant assays of *Frankenia thymifolia* aerial parts and root fractions (crude extract; hexane fraction; dichloromethane fraction; EtOAc fraction; BuOH fraction; water fraction).

	TPC (mg of GAE/g)	DPPH (mg of TE/g)	ABTS (mg of TE/g)	ORAC (mg of TE/g)	MCA (mg of EDTA/g)
Aerial parts					
Methanolic extract	87 ± 7^c^	132 ± 21^c^	154 ± 11^e^	305 ± 40^c^	16 ± 2^c^
Hexane	13 ± 4^e^	37 ± 4^e^	88 ± 3^f^	63 ± 18^e^	26 ± 3^b^
Dichloromethane	75 ± 5^d^	86 ± 17^d^	211 ± 20^d^	324 ± 51^c^	24 ± 1^b^
Ethyl acetate	221 ± 11^a^	282 ± 22^a^	778 ± 27^a^	918 ± 84^a^	39 ± 2^a^
Butanol	160 ± 7^b^	235 ± 15^b^	604 ± 36^b^	498 ± 48^b^	25 ± 5^b^
Water	83 ± 7^c^	136 ± 27^c^	321 ± 43^c^	174 ± 14^d^	26 ± 3^b^
Roots					
Methanolic extract	216 ± 11^b^	421 ± 61^b^	792 ± 64^b^	386 ± 57^bc^	16 ± 2^b^
Hexane	55 ± 9^f^	67 ± 13^c^	120 ± 10^e^	463 ± 43^b^	1 ± 1^e^
Dichloromethane	111 ± 12^e^	125 ± 17^c^	264 ± 34^d^	468 ± 57^b^	5 ± 2^d^
Ethyl acetate	308 ± 6^a^	821 ± 74^a^	1320 ± 63^a^	713 ± 100^a^	22 ± 2^a^
Butanol	152 ± 8^d^	420 ± 87^b^	697 ± 40^c^	303 ± 61^c^	14 ± 2^c^
Water	175 ± 7^c^	430 ± 82^b^	797 ± 36^b^	420 ± 59^b^	17 ± 2^b^

^a–f^Significant difference at *P* < 0.05 by Tukey's test.

**Table 2:** Phenolic compounds detected in ethyl acetate fraction by HPLC-DAD-ESI-MS from aerial parts and roots of *F. thymifolia* in negative mode.

Peak number	RT (mn)	*λ*_max_ (nm)	[M − H]^−^	Fragments	Organ	Tentative Identification
**1**	2.3	275	169	125	R	Gallic acid
**2**	3	310	153	123	AP	Hydroxytyrosol
**3**	3.9	280/310	137	93	AP	Hydroxybenzoic* p-*acid
**4**	3.9	280	609	483-441-423-305	R	Prodelphinidin B-4
**5**	4.2	280	635	465	R	Trigalloyl hexoside
**6**	4.5	280	333	289-265	AP	Posthumulone
**7**	4.5	280	449	287-431	R	Luteolin 7-*O*-glucoside
**8**	5.3	260/360	463	301, 178, 151	AP	Hyperoside
**9**	5.5	260/360	357	329	AP	Pinoresinol
**10**	5.5	290/320	497	344-402-449-482	R	Unknown ellagitannin
**11**	5.8	270/360	447	285	AP	Kaempferol 3-*O*-glucoside
**12**	6.7	290/320	312	135-178	AP	Feruloyl glycoside
**13**	6.7	285/320	655	378-484-543-587-619	R	ND
**14**	7.1	255/360	423	343-80	AP/R	Flavonoid sulfate
**15**	7.3	260/370	449	403-311-170	AP	Eriodictyol hexoside

**Table 3:** Pearson's correlation coefficients of antioxidant activities and total phenolic contents^a^.

	DPPH	ABTS	ORAC	MCA
Aerial parts				
TPC	0.9842^*∗∗∗*^	0.961^*∗∗*^	0.9594^*∗∗*^	0.6424^ns^
DPPH		0.96^*∗∗*^	0.9019^*∗*^	0.5918^ns^
ABTS			0.9008^*∗*^	0.7626^ns^
ORAC				0.6971^ns^
Roots				
TPC	0.9619^*∗∗*^	0.9734^*∗∗*^	0.5506^ns^	0.9445^*∗∗*^
DPPH		0.9935^*∗∗∗*^	0.5215^ns^	0.9456^*∗∗*^
ABTS			0.4754^ns^	0.9745^*∗∗∗*^
ORAC				0.3109^ns^

^a^Data represents Pearson's correlation coefficient *R*. ns indicates nonsignificant; *∗* refers to *P* < 0.05; *∗∗* and *∗∗∗* indicate significant at *P* < 0.01 and *P* < 0.001, respectively.
